# Calponin 3 Regulates Cell Invasion and Doxorubicin Resistance in Gastric Cancer

**DOI:** 10.1155/2019/3024970

**Published:** 2019-02-18

**Authors:** Keun-Seok Hong, Hyemin Kim, Seon-Hee Kim, Minju Kim, Jiyun Yoo

**Affiliations:** ^1^Division of Applied Life Science (BK21 Plus), Research Institute of Life Sciences, Gyeongsang National University, Jinju 52828, Republic of Korea; ^2^Division of Life Science, College of Natural Sciences, Gyeongsang National University, Jinju 52828, Republic of Korea

## Abstract

Calponin 3 (CNN3) is an F-actin-binding protein that regulates actin cytoskeletal rearrangement. However, the role of CNN3 in cancer cell invasion and resistance to chemotherapeutic agents has not yet been investigated. The present study was undertaken to investigate whether CNN3 influences cancer-related phenotypes in gastric cancer. We demonstrate that CNN3 contributes to cell invasion and resistance to doxorubicin in gastric cancer. CNN3 expression was markedly elevated in highly invasive cancer cell lines compared to less invasive or noninvasive cancer cell lines. Depletion of CNN3 protein suppressed the invasive ability of gastric cancer cells. The highly invasive MKN-28 gastric cancer cells were more resistant to doxorubicin than the noninvasive MKN-45 cells; however, knockdown of CNN3 expression in MKN-28 cells resensitized them to doxorubicin treatment. Taken together, our results suggest that CNN3 plays a key role in invasiveness and doxorubicin resistance in gastric cancer cells.

## 1. Introduction

Gastric cancer (GC) is the fifth most common cancer and the third leading cause of cancer-related mortality worldwide [1, 2]. Although the incidence of GC and mortality associated with this disease has gradually decreased in Japan and Korea, it still remains the second leading cause of death in Korea [3]. Metastasis is a complex, multistep process that requires the cancer cells to acquire several novel phenotypes, including invasion from the primary tumor through the extracellular matrix, intravasation, arrest, and extravasation from the circulatory system, followed by angiogenesis and growth at a distant site [4]. Despite advancements in our understanding of cancer mechanisms and improvement in cancer treatments over the last decade, metastasis remains the major cause of mortality in cancer patients. A mechanistic understanding of the metastatic process is essential for identifying novel molecular targets and developing therapies that are more effective.

Chemotherapy has been recognized as an effective and frequently used therapeutic method for advanced GC with or without metastasis [5]. Doxorubicin (Dox) is a member of the anthracycline family of drugs and, along with other chemotherapy agents, such as mitomycin and 5-fluorouracil, constitutes the gold standard treatment in advanced GC patients [6]. However, treatment based on Dox has a number of adverse effects, which lead to poor survival of GC patients [7, 8]. Chemotherapy drug resistance serves as the main contributor to treatment failure, bringing about tumor relapse and metastasis [9]. Although the mechanisms leading to this resistance are not fully established, increased drug efflux via overexpression and increased activity of multidrug resistance pumps, such as P-glycoprotein (P-gp), are well known [10–14]. Unfortunately, drug efflux pump inhibitors like cyclosporin A, ketoconazole, and verapamil add to the toxic side effects associated with doxorubicin treatment, thus decreasing the quality of life of cancer patients [15]. Therefore, Dox must be coadministered with a chemotherapeutic agent that abrogates doxorubicin resistance and has no overlapping benefits or side effects.

To identify the genes that are important for metastatic ability and drug resistance of GC cells, we compared the mRNA expression profiles of MKN-45, a noninvasive and drug-sensitive cell line, and MKN-28, a highly invasive and drug-resistant cell line. Among the genes differentially expressed between these two cell lines, we selected calponin 3 (CNN3) for further analysis because it was previously implicated in invasive properties of many cells [16, 17]. In this study, we found a significant correlation between CNN3 expression and cancer cell invasiveness in gastric and breast cancer (BC) cells and we demonstrated that CNN3 can positively regulate invasiveness and doxorubicin resistance in GC cells.

## 2. Materials and Methods

### 2.1. Cell Cultures and Reagents

Human gastric cancer cell lines MKN-45, MKN-28, SNU-484, SNU-638, and SNU-719 were obtained from the Korean Cell Line Bank (Seoul, Korea). Human breast cancer cell lines SK-BR-3, MDA-MB-435, MDA-MB-231, and MCF-7 were purchased from the American Type Culture Collection (ATCC). The human gastric cancer cell lines were maintained in RPMI 1640 (Life Technologies), and breast cancer cell lines were maintained in a DMEM medium (Life Technologies) supplemented with 10% fetal bovine serum and antibiotics. Doxorubicin was purchased from Sigma-Aldrich.

### 2.2. Total RNA Extraction and Reverse Transcription-Polymerase Chain Reaction (RT-PCR)

Total RNA was extracted from the cultured cells using the RNeasy Mini Kit (QIAGEN, Hilden, Germany). RT-PCR was performed using a Maxime RT-PCR PreMix Kit (Intron, Taejon, Korea). Total RNA (200 ng) and specific primers were added into the Maxime RT-PCR PreMix tubes, and RNase-free water was added to a total volume of 20 *μ*l. RT-PCR was performed using a Thermo Electron PCR thermal cycler. Amplified products were separated on 1.5% agarose gels containing 0.1 *μ*g/ml ethidium bromide. The following primers were used to amplify most of the coding region of CNN3 (sense, 5′-TCCCAAAATGCAAACTGACA-3′, and antisense, 5′-CCGAACCATTTGTTCCTGTT-3′) and *β*-actin (sense, 5′-GTGGGGCGCCCCAGGCACCA-3′, and antisense, 5′-CTCCTTAATGTCACGCACGAT-3′).

### 2.3. Antibodies and Western Blot Analysis

Rabbit anti-CNN3 antibody was purchased from Proteintech Group. Rabbit anti-PARP antibody was from Cell Signaling Technology. Mouse anti-alpha-tubulin antibody was from Sigma-Aldrich. For western blot analysis, cells were harvested after a fixed period of time and lysed in lysis buffer containing Tris (20 mM), EDTA (2 mM), sodium chloride (150 mM), sodium deoxycholate (1 mM), Triton X-100 (1%), glycerol (10%), and 2 pills of protease inhibitor cocktail on ice for 1 h, then centrifuged at 13,000 rpm for 15 min. Cell lysates were separated by SDS-PAGE and transferred to Immobilon-P membrane (Millipore Co., USA). Subsequently, the membrane was incubated at room temperature for 1 h in TBST solution containing Tris–HCl (10 mM, pH 8.0), NaCl (150 mM), and Tween 20 (0.05%) supplemented with nonfat dry milk (5%) and probed with the appropriate primary antibodies at 4°C for 12 h. The bound antibody was visualized with a suitable secondary antibody conjugated with HRP (horseradish peroxidase), using enhanced chemiluminescence reagents (ECL, Amersham Bioscience). For normalization, *α*-tubulin expression was used as a control.

### 2.4. RNA Interference Experiments

Two different siRNA oligo duplexes for targeting the *CNN3* gene were purchased from Bioneer (Daejeon, Korea). The sequence was as follows: siCNN3-1: 5′-GAAACAUGACCCAGGUUCA-3′, siCNN3-2: 5′-CCUGUUUGUGCCAAUGUAU-3′, and siCon: 5′-AAUCGCAUAGCGUAUGCCG-3′. Each siRNA oligo duplex was transiently transfected by using siLentFect Lipid Reagent (Bio-Rad) according to the manufacturer's instructions. 48 h after incubation, the efficiency of the CNN3 downregulation by each siRNA oligo duplex was confirmed by western blotting using the anti-CNN3 antibody.

### 2.5. Migration and Invasion Assays

Migration and invasion assays were performed as described previously [18]. For wound-healing assays, 4.0 × 10^4^ cells in 70 *μ*l of medium were seeded into Culture-Insert (ibidi). After the cells were confluent, they were pretreated with mitomycin C (10 *μ*g/ml, Sigma-Aldrich) for 2 h to inhibit the effect of cell proliferation and washed with culture medium. After removal of the Culture-Insert, the cells were incubated with appropriate fresh media and photos were taken using a phase-contrast microscope at 0 and 24 h. Cell invasion assays were performed using the QCM 24-Well Cell Invasion Assay Kit (CHEMICON). Cells (70% confluency) were grown in six-well plates and starved with serum-free media for 18 h. The starved cells were resuspended again in 1 ml of serum-free media. Aliquots of 250 *μ*l of cell suspension (1 × 10^6^ cells/ml) were added to each insert, and 500 *μ*l of appropriate fresh media containing chemoattractant (20% FBS) was added to the lower chamber. The invasion chamber insert containing invaded cells was transferred to a clean well containing prewarmed Cell Detachment Solution (225 *μ*l) and incubated at 37°C for 30 min. The insert was removed from the well. Each well received 75 *μ*l of lysis buffer/dye solution and was incubated at room temperature for 15 min. Then, 200 *μ*l of the mixture was transferred to a 96-well plate and evaluated with a fluorescence plate reader using a 480/520 nm filter set.

### 2.6. Proliferation Assay

The proliferation assay was performed as described previously [18]. The 2 × 10^4^ cells were transferred to a 6-well plate. 1 to 4 days after incubation, the cells were trypsinized and resuspended in an appropriate medium. Cell suspensions were centrifuged, and the cell pellets were resuspended again in an appropriate medium. The viable cells were stained with trypan blue and counted with a hemocytometer.

### 2.7. Cell Viability and Apoptosis Detection

The cell proliferation reagent MTS (Promega) was used to determine cell viability. The MTS assay was then performed according to the manufacturer's instruction at 48 h after treatment with doxorubicin at indicated concentrations. The In Situ Cell Death Detection Kit, Fluorescein (Roche) was used to observe the morphology of apoptotic cell death. Cells treated with doxorubicin for 48 h were washed with cold PBS and fixed with paraformaldehyde (4%). After permeabilization, cells were stained with the TUNEL reaction mixture in the dark. The cells were also stained with DAPI solution (1 *μ*g/ml) in the dark at room temperature for 5 min and observed under a fluorescence microscope. The rate of apoptosis was quantified by the rate of TUNEL-positive cells.

### 2.8. Statistical Analysis

Statistical analyses were performed using the unipolar, paired Student *t*-test. *P* values < 0.05 were considered as statistically significant.

## 3. Results

### 3.1. CNN3 Expression Is Correlated with Invasiveness of Cancer Cell Lines

**Figure 1:**
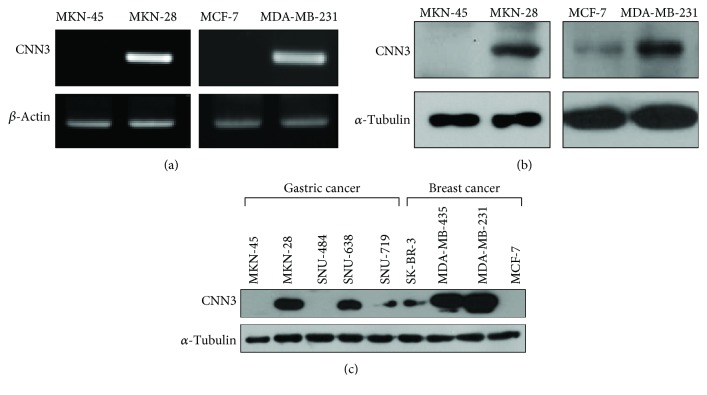
Correlation between CNN3 expression and cancer cell invasiveness. (a, b) RT-PCR and western blot analysis were performed to analyze the expression level of CNN3 mRNAs (a) and proteins (b) in human gastric cancer cells (MKN-45 and MKN-28) and breast cancer cells (MCF-7 and MDA-MB-231). (c) Basal expression of CNN3 in different human gastric cancer and breast cancer cell lines. Different human gastric cancer cell lines (MKN-45, MKN-28, SNU-484, SNU-638, and SNU-719) and breast cancer cell lines (SK-BR-3, MDA-MB-435, MDA-MB-231, and MCF-7) were harvested, and western blot analyses were performed to analyze the expression of CNN3 and *α*-tubulin.

To determine whether the expression of CNN3 is correlated with the invasiveness of GC cells, we first compared the expression levels of CNN3 in MKN-45 and MKN-28 cells, which are known to be noninvasive and highly invasive GC cell lines, respectively. Interestingly, the mRNA expression of CNN3 was markedly increased in MKN-28 cells compared to the MKN-45 cells (Figure 1(a), left). We next tested whether the mRNA expression of CNN3 was also elevated in other invasive cancer cell lines. We found that the highly invasive BC cell line MDA-MB-231 has higher levels of CNN3 mRNA compared to the noninvasive BC cell line MCF-7 (Figure 1(a), right). Western blot analysis also showed consistent expression of CNN3 proteins in these four cell lines (Figure 1(b)). Among the various GC cell lines, MKN-28 and SNU-638 cells have been known to be more invasive than MKN-45, SNU-484, and SNU-719 cells. MDA-MB-435 and MDA-MB-231 BC cells are also more invasive than MCF-7 and SK-BR-3 cells. Thus, we examined whether CNN3 expression is elevated in highly invasive cancer cell lines compared to the less or noninvasive cancer cell lines of the same cancer type. As shown in Figure 1(c), the expression of CNN3 is strikingly increased in invasive cancer cell lines when compared to the noninvasive cancer cell lines. These results indicate that there is a significant correlation between CNN3 expression and cancer cell invasiveness and increased expression of CNN3 could influence invasiveness of various cancer cells.

### 3.2. Depletion of CNN3 Expression Suppresses GC Cell Migration and Invasion

**Figure 2:**
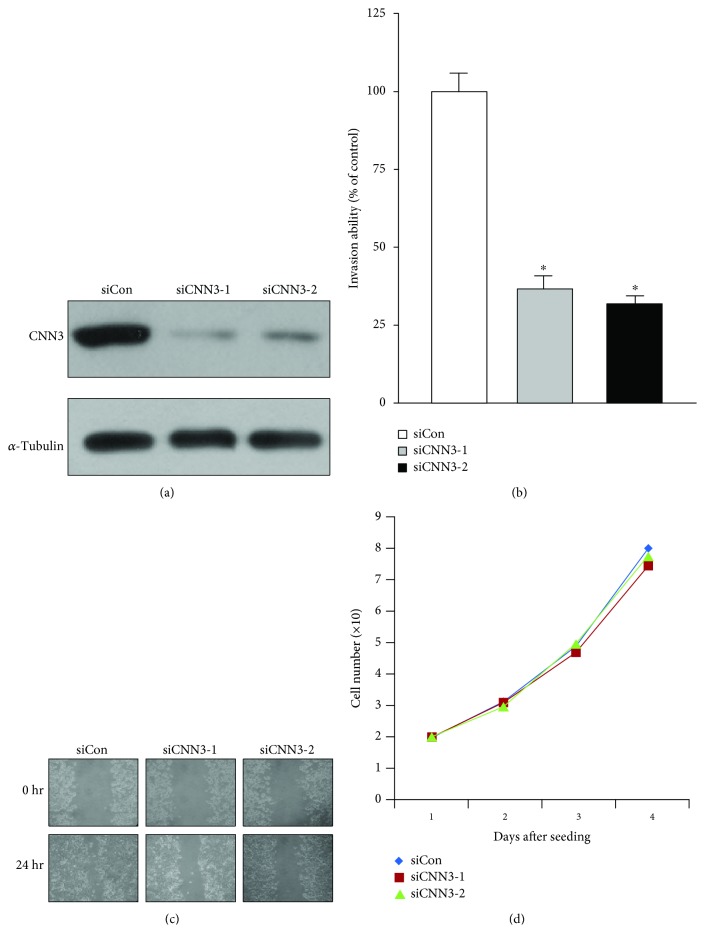
CNN3 is important for the invasiveness of MKN-28 gastric cancer cells. (a) MKN-28 cells were transfected with control (siCon) or two different CNN3-specific siRNAs (siCNN3-1 and siCNN3-2) using siLentFect Lipid Reagent. Western blot analysis was performed against indicated antibodies. (b) Control siRNA- and CNN3 siRNA-transfected MKN-28 cells were seeded onto Matrigel matrix-coated upper chambers. The invasion activity of each cell was represented as % of control cells. The data shown are expressed as the means ± SD of three individual experiments, each performed in triplicate. ^∗^*P* < 0.01 as determined by *t*-test. (c) Control siRNA- and CNN3 siRNA-transfected MKN-28 cells were analyzed in wound-healing assays by visualizing wound closure via phase-contrast microscopy. (d) Effect of CNN3 depletion on the proliferation of MKN-28 cells.

In an effort to determine whether CNN3 expression is associated with invasive properties of GC cell lines, we assessed the effects of CNN3 depletion on cancer cell migration and invasion in MKN-28 cells. To accomplish this, MKN-28 cells were transfected with two different CNN3-specific siRNAs and the efficacy of siRNA-based knockdown was determined by western blotting. As shown in Figure 2(a), the protein level of CNN3 was markedly reduced by the respective siRNAs, but not by the control siRNA. To determine the effect of CNN3 depletion on cancer cell invasion, we performed an *in vitro* two-chamber invasion assay. As shown in Figure 2(b), depletion of CNN3 expression markedly suppressed the invasive ability of MKN-28 cells (siCNN3-1 and siCNN3-2) compared with that of control cells (siCon). Next, we checked the migration ability of CNN3-depleted MKN-28 cells by using a wound-healing assay. Twenty-four hours after wounding, complete wound closure was achieved in the MKN-28 control cells, whereas wound closure was significantly inhibited in the CNN3-depleted MKN-28 cells (Figure 2(c)). Taken together, these results suggest that upregulation of CNN3 expression plays a key role in GC cell migration and invasion. To exclude the possibility that the effect of CNN3 on the migration and invasion of MKN-28 GC cells was attributable to different proliferation rates, the growth rates of CNN3-depleted MKN-28 cells (siCNN3-1 and siCNN3-2) were compared with those of control (siCon) cells. Under the same growth conditions, all cells showed similar growth rates (Figure 2(d)), suggesting that the decreased migration and invasion in CNN3-depleted MKN-28 cells (siCNN3-1 and siCNN3-2) were not associated with the proliferation rate.

### 3.3. CNN3 Is Required for Doxorubicin Resistance in MKN-28 Cells

**Figure 3:**
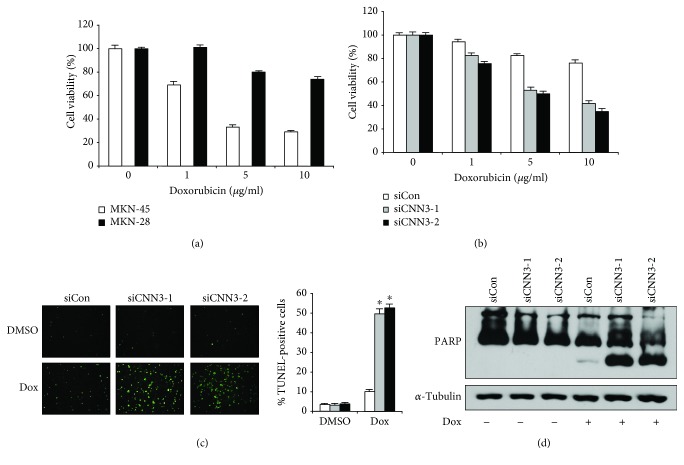
CNN3 is critical for doxorubicin resistance in MKN-28 cells. (a) Viability of two different human gastric cancer cell lines (MKN-28 and MKN-45) after treatment with the indicated concentrations of doxorubicin for 48 h. (b) Viability of control siRNA- and CNN3-specific siRNA-transfected MKN-28 cells after treatment with the indicated concentrations of doxorubicin for 48 h. (c) Representative images for TUNEL staining of CNN3-depleted MKN-28 cells after doxorubicin (Dox) treatment (2 *μ*g/ml) for 24 h. The histogram shows the ratio of TUNEL-positive CNN3-depleted MKN-28 cells after Dox treatment (2 *μ*g/ml) for 24 h. The data shown are expressed as the means ± SD of three individual experiments, each performed in triplicate. ^∗^*P* < 0.01 as determined by *t*-test. (d) Representative immunoblot for PARP cleavage in control siRNA- and CNN3-specific siRNA-transfected MKN-28 cells after Dox treatment for 48 h.

It is generally known that invasive cancer cells are resistant to many chemotherapeutic agents [19]. We confirmed that Dox treatment markedly decreases the viability of MKN-45 cells in a dose-dependent manner, whereas MKN-28 cells are significantly more resistant to Dox than MKN-45 cells (Figure 3(a)). Since increased CNN3 expression is critical for invasiveness of MKN-28 cells, we tested whether CNN3 plays an important role for Dox resistance in MKN-28 cells. To determine the effect of CNN3 expression on Dox resistance, we checked the viability of CNN3-depleted MKN-28 cells after Dox treatment by MTS assay. As shown in Figure 3(b), the viability of CNN3-depleted MKN-28 cells was markedly decreased after Dox treatment as compared to that of the control cells. To determine whether the depletion of CNN3 expression affects Dox-induced apoptosis, we analyzed the cells by TUNEL staining. As shown in Figure 3(c), depletion of CNN3 significantly increased Dox-induced apoptosis in MKN-28 cells compared to the control cells. Consistent with these results, depletion of CNN3 increased Dox-induced cleavage of poly (ADP-ribose) polymerase (PARP), a substrate of activated caspase during apoptosis, in MKN-28 cells (Figure 3(d)). Taken together, these results suggest that upregulation of CNN3 expression confers resistance to Dox-induced apoptosis in GC cells.

## 4. Discussion

CNN3 phosphorylation by Rho-associated protein kinase (ROCK) during myogenesis is known to regulate actin cytoskeleton rearrangement [20] and stress fibers of dermal fibroblasts during wound healing [21]. Since reorganization of the actin cytoskeleton is a primary mechanism of cell motility and essential for most types of cell migration [22], it is possible that increased CNN3 promotes migration of GC cells. CNN3 constitutively associates with both ERK1/2 and PKC alpha and indirectly stimulates phosphorylation of ERK1/2 by PKC alpha. Phosphorylated ERK1/2 translocates to actin filaments, where it binds and phosphorylates its substrate, Cad [23]. Activated Cad is also involved in regulating breast cancer cell migration and invasion [24]. Moreover, CNN3 phosphorylation leads to phosphorylation of p38 to promote trophoblast invasion [17]. Like ERK1/2, p38 can interact with actin filaments and regulate their dynamics. Thus, CNN3 is thought to play a pivotal role in activation of signaling molecules related to the migration of cancer cells. Consequently, there is a close relationship between CNN3 and cell migration. Since cell migration is an essential step in tumor invasion and metastasis, it is possible that increased CNN3 expression can promote invasion of cancer cells by reorganization of the actin cytoskeleton or by activation of other molecules. Based on these studies, CNN3 has been known to be involved in trophoblast invasion [17] and has been identified as a marker for lymph node metastasis in colorectal cancer [16]. Recently, it has also been suggested that the higher CNN3 expression may play a role in increasing colorectal cancer cell invasion partially due to its association with the lower E-cadherin level [25]. The mechanism of CNN3 to decrease the E-cadherin level should be further investigated, but these results strongly support our findings that CNN3 positively regulates the invasiveness of GC cells.

Multidrug resistance (MDR) is a principal cause for the failure of many forms of chemotherapy. Usually, invasive cancer cells exhibit higher resistance to anticancer drugs than noninvasive cancer cells. Dox is one of the frequently used anticancer drugs for GC patients [6]. We found that CNN3 expression levels are increased in MKN-28 compared to MKN-45 cells, and CNN3 knockdown in MKN-28 cells resensitizes them to Dox treatment. There is no direct connection between CNN3 and drug resistance; however, changes in the actin cytoskeleton of MDR cell lines have been documented, and CNN3 regulates the actin cytoskeleton. In MDR osteosarcoma cells, the number of well-organized actin stress fibers was increased, but in parental cells, actin filaments were diffusely spread throughout the cytoplasm [26]. Another actin-binding protein, twinfilin 1, regulates chemoresistance in breast cancer cells by promoting the epithelial-to-mesenchymal transition (EMT) [27]. Cancer cells with increased EMT exhibit high chemoresistance [28]. Therefore, further investigation is required to determine whether increased invasion and chemoresistance mediated by CNN3 is a result of increased EMT.

In summary, in the present study, we suggested for the first time that CNN3 can directly contribute to GC invasion and resistance to chemotherapeutic agents such as doxorubicin. Thus, CNN3 contributes to tumor progression by inducing multiple changes in cellular physiology. In this context, molecular targeting of CNN3 may not only prevent the seeding of GC cells to other organs but also sensitize cancer cells to chemotherapy, thereby preventing the spread of GC.

## Data Availability

The data used to support the findings of this study are available from the corresponding author upon request.
